# EBR and JA regulate aroma substance biosynthesis in ‘Ruidu Hongyu’ grapevine berries by transcriptome and metabolite combined analysis

**DOI:** 10.3389/fpls.2023.1185049

**Published:** 2023-06-06

**Authors:** Jiajia Li, Yi Quan, Zishu Wu, Jiayu Han, Ying Zhang, Hafiz Umer Javed, Chao Ma, Songtao Jiu, Caixi Zhang, Lei Wang, Shiping Wang

**Affiliations:** ^1^ Department of Plant Science, School of Agriculture and Biology, Shanghai Jiao Tong University, Shanghai, China; ^2^ Grape and Wine Institute, Guangxi Academy of Agricultural Sciences, Nanning, Guangxi, China; ^3^ College of Chemistry and Chemical Engineering, Zhongkai University of Agricultural Engineering, Guangzhou, China

**Keywords:** grapevine, EBR, JA, HS-SPME/GC−MS, aroma, ‘Ruidu Hongyu’, transcriptome sequencing

## Abstract

Volatile compounds including terpenes, aldehyde, phenol, and alcohol are significantly contributed floral and fruity aromas to the Muscat variety. ‘Ruidu Hongyu’ grapevine is one of the newly developed grape varieties, and cultivation of this variety has been extended across China due to unique quality traits and taste. In this study, HS-SPME/GC−MS and transcriptome sequencing analysis were performed to evaluate the impact of exogenous 2,4-epibrassinolide (EBR), jasmonic acid (JA), and their signaling inhibitors brassinazole (Brz)/sodium diethyldithiocarbamate (DIECA) on the biosynthesis of aroma substances in ‘Ruidu Hongyu’ grapevine. According to the results, exogenous BR and JA promoted the accumulation of various aroma substances, including hexenal, 2-hexenal, nerol oxide, vanillin, hotrienol, terpineol, neral, nerol, geraniol, and geranic acid. After EBR and JA treatments, most of the genes responsible for terpene, aldehyde, and alcohol biosynthesis expressed at a higher level than the CK group. Relatively, EBR treatment could not only promote endogenous BR biosynthesis and metabolism but also elevate BR signaling transduction. JA treatment contributed to endogenous JA and MeJA accumulation, as well. Through transcriptome sequencing, a total of 3043, 903, 1470, and 607 DEGs were identified in JA vs. JD, JA vs. CK, BR vs. CK, and BR vs. Brz, respectively. There were more DEGs under both EBR and JA treatments at late fruit ripening stages. The findings of this study increase our understanding regarding aroma substances biosynthesis and endogenous BR/JA metabolism in response to exogenous EBR and JA signals.

## Introduction

Aroma is one of the most eminent indices to gauge grape wine quality (Pinu et al., 2019). Though the overall composition of most wine grape varieties is quite similar, the differences in aroma composition and flavor between them are quite remarkable (Ruiz et al., 2019). The aroma of high-quality wines is usually rich, balanced, and typically outstanding (Rinaldi et al., 2020). There are eight categories represented volatile compounds in grape berries, namely monoterpenes, norisoprenoids, aliphatics, higher alcohols, esters, phenylpropanoids, and methoxypyrazines (Wu et al., 2019) and they mainly contribute to the fragrance of flowers, citrus, tropical fruit, violet, and green pepper, respectively, which together constituted the complex and elegant aroma of high-quality wine (Richmond et al., 2019). Terpenes are one of the most important components of aroma substances, which exist as free and combined forms in wine grapes. Because of its low threshold and great contribution to the grape aroma, it has been widely studied (Jin et al., 2022). The majority of terpenes found in wine grapes are monoterpenes, including linalool, geraniol, citronellol, nerol, *α*-terpinol, menthol and citral (Chigo-Hernandez et al., 2022), which have a significant impact on the aroma profile of the wine (Yue et al., 2022). Compared with monoterpenoids, sesquiterpenes are present at lower levels in grape berries and wine but they play a vital role in wine aroma (Luo et al., 2019). Moreover, alcohols, esters, and aldehydes all play a significant role in the aroma composition of mature grape berries. Furthermore, different compounds have different sensory thresholds and their interaction impart the characteristic flavor to grapevines (Wu et al., 2020).

Till now, the pathways of terpenes biosynthesis and metabolism have been systematically paraphrased (Englund et al., 2018). Plant terpenes are mainly synthesized independently by two pathways (Qiao et al., 2021). The mevalonic acid (MVA) pathway located in the cytoplasm, and the 1-deoxy-D-xylulose 5-phosphate (DXP)/methylerythritol phosphate (MEP) pathways located in the plastid (Zhang et al., 2021). In MVA pathway, there are seven rate-limiting enzymes, including acetyl-CoA C-acetyltransferase (AACT), 3-hydroxy-3-methylglutaryl-CoA synthetase (HMGS), 3-hydroxy-3-methylglutaryl coenzyme-A reductase (HMGR), phosphomevalonate kinase (PMK), geranyl diphosphate synthase (GPPS), farnesyl diphosphate synthase (FPPS) and terpene synthases (TPS) (Mukherjee et al., 2022). Moreover, A total of seven key rate-liming enzymes are responsible for participating in the MEP pathway, namely Deoxy-D-xylulose 5-phosphate synthase (DXS), DXP reducto-isomerase (DXR), 2-C-methyl-D-erythritol 4-phosphate cytidylyltransferase (MCT), 4-hydroxy-3-methylbut-2-en-1-yl diphosphate synthase (HDS), geranyl geranyl pyrophosphate synthase (GGPPS), GPPS and TPS (Wan et al., 2021). After a series of enzymatic reactions, sesquiterpene and geranyl linalool are finally biosynthesized in the cytoplasm, while diterpene and monoterpene are biosynthesized in the plastid (Lv et al., 2022b).

The accumulation of aroma substances could be regulated by many factors, including the environment, the cultivation pattern, and exogenous plant growth regulators (Kou et al., 2021; Yang et al., 2022). Recently, brassinolide (BR), was recognized as the sixth-largest phytohormone due to its promoting effects on the formation of multiple biochemical properties in grapevine (*Vitis vinifera* L.) (Li et al., 2023). In terms of terpenes contents, 100 μmol L^-1^ BR was reported to increase *β*-pinene and *D*-limonene in ‘Kyoho’ grape berries. Furthermore, 3-hydroxy-3-methyl glutaryl coenzyme A reductase (HMGR) activity was increased by the induction of exogenous BR (Zheng et al., 2021). It is noteworthy that MeJA also had a great impact on the increment of aroma compounds such as monoterpenes in ‘Muscat Hamburg’ grape pericarp, especially linalool, α-terpineol, and oxides (Yue et al., 2021). Furthermore, the application of MeJA was also responsible to raise the content of 1-hexanol, hexanal, and 2-heptanol in ‘Cabernet Sauvignon’ grapevine. Overall, BR and JA both played enormous roles in aroma substance accumulation in the grapevine. However, further research work needs to be conducted specifically for the understanding of the molecular mechanism.

‘Ruidu Hongyu’ grapevine, as a table grape cultivar in China, has various advantageous traits including high-yield, pink coloring and precocity (Xu et al., 2014). Previously, the effect of EBR and JA on berry quality was evaluated, and they were responsible for improving anthocyanin, fatty acid, and sugar levels (Li et al., 2022). However, the mechanism of exogenous BR and JA regulation on aroma volatilization of ‘Ruidu Hongyu’ grapevine has yet to be addressed. Therefore, a co-analysis of metabolite and transcriptome was conducted in this study. After that, we assessed how EBR, JA, and their signaling inhibitors (Brz and DIECA), influenced the aroma substances biosynthesis in the ‘Ruidu Hongyu’ grapevine. Moreover, a molecular study at the transcriptional level was implemented to understand the potential mechanism. Above findings aimed to suggest exogenous EBR or JA application was a suitable strategy to improve the flavor of table grapes, which could be carried out in future grapevine cultivation.

## Materials and methods

### Vineyard, EBR and JA treatments, and sampling set

Six-year-old ‘Ruidu Hongyu’ grapevine was cultivated in the Grape and Wine Institute, Guangxi Academy of Agricultural Sciences (22.61°N, 108.24°E, Nanning, Guangxi, China) in 2021. In this study, five treatments group such as the CK, 0.5 mg L^-1^ EBR (BR), 0.5 mg L^-1^ EBR + 1 mg L^-1^ Brz (BR signaling inhibitor; BB), 100 μmol L^-1^ JA, and 100 μmol L^-1^ JA + 10 mmol L^-1^ DIECA (JA signaling inhibitor; JD) were selected to evaluate the aroma and their mechanism in ‘Ruidu Hongyu’ grapevine. Applications were administered to the grape berries at DAA 30 (days after anthesis), and three sampling intervals—DAA 30 (0 days after treatment), DAA 50 (20 days after treatment), and DAA 70 (40 days after treatment) were defined. A total of 300 berries were collected from 20 vines of each treatment and CK group at every sampling stage. All berries were immediately transported with dry ice to the Department of Plant Science, School of Agriculture and Biology, Shanghai Jiao Tong University (121°29’ W, 31°11’ N, Shanghai, China). Furtherly, berry size and berry weight were determined by a vernier caliper (Mitutoyo, TKY, Japan) and an analytical balance (Sartorius, German), respectively. Then, grape berries were ground using a vacuum grinder (IKA, GER). Powder was frozen under liquid nitrogen and stored at −80°C for further analysis, including the determination of free-form aroma substances, RNA extraction and qRT-PCR analysis.

### Chemicals

Chemicals used for the detection of VOCs including D-gluconolactone and 4-methyl-2-pentanol were purchased in Sigma-Aldrich (SHH, CHN), and PVPP, NaCl used for extraction of VOCs were purchased in Sangon Biotech (SHH, CHN). Moreover, TB Green^®^ Fast qPCR Mix used for qRT-PCR was purchased in TaKaRa (Dalian, China).

### Free-form volatiles detected by HS-SPME/GC−MS

The previously established method of our research group (Li et al., 2017; Javed et al., 2018; Javed et al., 2019) for the determination of free-form volatiles was applied in this study. Approximately 100 grape berries without seeds from each treatment group were crushed, and 1 g each of polyvinylpyrrolidone (PVPP) and D-gluconolactone were added. The powder was naturally thawed at a 4°C (refrigerator) for 4 h, followed by a 10 min centrifugation process at 4°C with 8,000 *g*.

Next, took 5 mL grape juice supernatant to a 15 mL vial containing 1 g of sodium chloride (NaCl) and then 10 μL of 4-methyl-2-pentanol (internal standard, concentration was 1.0018 g L^-1^) was mixed. The vial was equilibrated at 40°C for 30 min with agitation at 500 rpm. Before sample extraction, activated 2 cm DVB/CAR/PDMS 50/30 μm solid-phase micro-extraction (SPME) fiber (Supelco, Bellefonte, PA, USA) at 250°C. Following that, under the same agitation condition, the activated SPME fiber was inserted into the headspace at 40°C for 30 min. In the end, inserted it into the GC injector port for 8 min to desorb the volatiles.

The determination of volatile compounds in grape juice was carried out on an Agilent 7890 gas chromatography (GC) system (Santa Clara, CA, USA), with an autosampler system in conjunction with an Agilent 5975C mass spectrometer (Santa Clara, CA, USA). Helium was equipped in the system and supplied at a flow rate of 1 mL min^-1^. The specific procedure was as follows: after maintaining the temperature at 40°C for five minutes at a rate of 5°C per minute, it was raised to 240°C at a rate of 20°C per minute. Finally, the temperature was raised to 260°C and held there for 5 minutes. Three replicates were set in this study, and the final value of each free-form volatile was reported in mg L^-1^ and shown in the heat map. The information and retention time of terpenes detected in this study was shown in **Table S2**.

### RNA extraction, illumina sequencing, and transcriptome data analysis

The RNA extraction of berry samples was carried out on an RNA prep Pure Plant Plus Kit (TaKaRa, Dalian, China). A BIO-RADXR gel imaging analysis system (Bio-Rad, CA, United States) was used to assess the quality and integrality of the extracted RNA. A total of 24 libraries were established using Illumina HiSeq X Ten (Illumina Inc., San Diego, CA, USA), and produced 150 bp paired-end reads. At least three biological replicates should be used in each treatment

The sequencing was performed at Shanghai APPLIED PROTEIN TECHNOLOGY (SHN, China). Trimming adaptor sequences were used to clean raw reads, and the clean reads were aligned in the Tophat v2.0.943 program, referring to a grapevine reference genome (https://www.ncbi.nlm.nih.gov/genome/?term=grape). The raw RNA sequencing data has been uploaded and deposited into the National Center for Biotechnology Information (NCBI) BioProject database, and the accession number was PRJNA943117.

In this research, the absolute value of the log2 (fold change) with fragments per kilobase million (FPKM) §1 was used as a threshold for the discrimination of the significant differentially expressed genes (DEGs) throughout the detection of DEGs. Based on the calculated FPKM values, DEGs between different samples were analyzed. The main screening steps were as follows: (1) DESeq software was used to standardize the number of gene counts in each sample, and different expression fold-changes were computed. (2) A negative binomial distribution test was carried out to determine the significance of different readouts. Two criteria were used to determine the differential expression of the same gene between the compared samples. One is based on the expression fold change, i.e. the fold change of the same gene expression level between two samples. The other is based on the FDR (false discovery rates) value, which is calculated by first calculating the p-value for each gene and then using the FDR error control method to correct the p-value for multiple hypothesis testing. The criteria for DEGs of the final screen were p < 0.05 and a fold change in expression greater than 2. GO (Gene Ontology) enrichment analysis was performed after obtaining DEGs. Gene ontology (GO) enrichment and KEGG pathway analysis were performed for the differentially expressed genes (DEGs), through an online website (http://cloud.aptbiotech.com/#/product-list).

### qRT-PCR determination

To further verify the sequencing results obtained from RNA sequencing, A PrimeScript™ RT Reagent Kit in combination with gDNA Eraser (Perfect Real Time) (TaKaRa, Dalian, China) was used to extract one microgram of total RNA. After that, the first-strand cDNA was obtained. The total final volume was 10 μl in total, made up of 5 μl of TB Green^®^ Fast qPCR Mix, 3 μl of ddH_2_O, 1 μl of a forward and reverse primer mixture, and 1 μl of cDNA. Then, qRT−PCR was carried out in a CFX-connected Real-Time PCR Detection System (Bio-Rad, CA, USA). The sequences of all genes and transcription factors identified in this study were acquired from the NCBI database (https://www.ncbi.nlm.nih.gov/). The primers were designed by the qPrimerDB-qPCR Primer Database (https://biodb.swu.edu.cn/qprimerdb/browse_plants). All primer sequence information was shown in **Table S1**. The following procedure was used to conduct the reactions: 95°C for 20 s, followed by 39 cycles of 95°C for 15 s, 55°C for 15 s, and 60°C for 15 s. The determination of relative gene expression was according to the 2^-ΔΔCt^ method.

### Statistical analysis

One-way ANOVA and Duncan’s multiple range test in SPSS version 19.0 (IBM Corp., Armonk, NY, USA), and the principal component analysis (PCA) were used for data analysis at the p ≤ 0.05 level. Heatmaps of metabolite concentrations as well as gene expressions were constructed by GraphPad Prism 9.0 (GraphPad Software, Inc., San Diego, CA, USA) and Visio 2020 (Microsoft, SEA, USA).

## Results and discussion

### Exogenous EBR and JA ameliorate physiological parameters of grape berries

Physiological parameters such as longitudinal diameter, transverse diameter, single berry weight, total soluble solid (TSS), and titratable acids (TA) were measured in CK, BR, JA, BB, and JD treatment groups throughout the grapevine development and ripening stages (**Figure S1**). Exogenous EBR and JA were found to be increased berry size and TSS while decreased TA contents (**Figures S1A, B**). Furthermore, lower TSS content was observed in the BB and JD applications at DAA 50 and DAA 70. TA levels were higher in both the BB and JD treatments throughout the study.

### Exogenous EBR and JA regulated aroma compounds formation of grape berries

**Figure 1:**
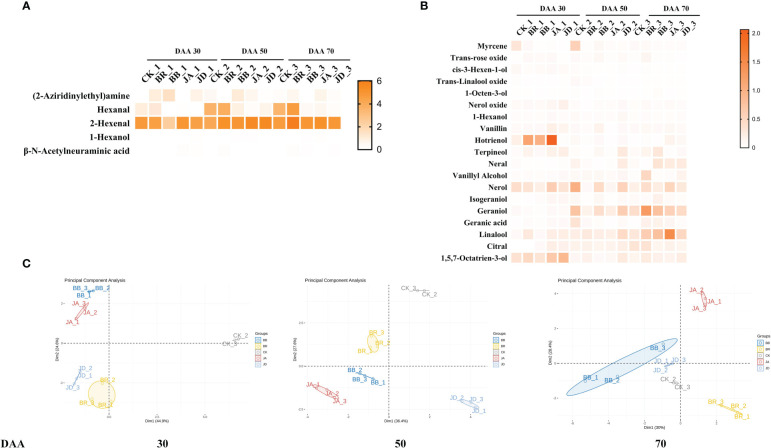
Effects of nutrient solution of different concentrations on grape berry free-form aroma volatiles formation. **(A)** Alcohol and aldehyde levels. **(B)** Terpenes levels. **(C)** PCA analysis. The contents of each aroma substance were shown as heatmap.

In this study, a total of 24 kinds of free-form compounds including 1 alcohol (1-Hexanol); 2 aldehydes (Hexenal, 2- Hexenal) and 19 terpenes were detected successfully. In all, BR and JA treatments have more VOCs (Volatile Organic Compounds) than the CK group. Furthermore, the concentration of free-form terpene, alcohol, and aldehyde increased under all treatments throughout the grapevine development and ripening. Especially at DAA 70, the increasing trend becomes noticeable (**Figures 1A, B**). Interestingly, the exogenous application of EBR at DAA 50 and DAA 70 and JA at DAA 70 increased the content of aldehydes (hexanal and 2-hexenal) and terpenes (hotrienol, terpineol, nerol, geraniol, linalool, and citral). Compared to the BR and JA groups, their contents dropped in both BB and JD treatments, respectively.

To further explore the variability between and within groups of treatments, the PCA analysis was implemented (**Figure 1C**). The results showed that the degree of parameter’s variation within each treatment group was not significant. However, the degree of parameter’s variation among the CK, BR, JA, BB and JD treatment groups was evident. Thus, we concluded that a high reproducibility was indicated within the groups, and good discrimination was shown between the groups.

**Figure 2:**
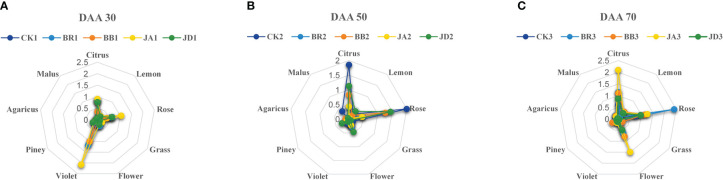
Aromatic series values of grape berries for the different developmental stages under EBR, JA, BB and JD treatments and CK treatments (CK) (n = 3). **(A)** Aromatic series values at DAA 30. **(B)** Aromatic series values at DAA 50. **(C)** Aromatic series values at DAA 70.

After EBR and JA treatment, we found that the concentration of alcohols, aldehyde, and terpenes compounds at fruit ripening stages (DAA 50 and 70) was higher than those in the CK group. Furthermore, a radar map was conducted to display the effect of EBR and JA treatment on the grape aroma. At DAA 30, we found that JA treatment contributed the most to violet aroma (**Figure 2A**). But at DAA 50, the citrus and rose aromas were hugely promoted by CK treatment (**Figure 2B**). Moreover, we noticed that BR treatment mainly contributed to rose aroma while JA treatment was responsible for potent citrus and floral aromas at DAA 70 (**Figure 2C**). Furthermore, BB and JD treatments had different degrees of inhibition effect on the formation of different aromas (**Figure 2**). For example, at the early fruit ripening stage (DAA 30), both BB and JD treatments inhibited the increasement of citrus and rose aromas (**Figure 2A**). In case of the late fruit ripening stage (DAA 50 and DAA 70), citrus and flower aromas were decreased when BB and JD application was applied on grapevine (**Figures 2B, C**).

### Transcriptome analysis of BR and JA treatments on grape berries

As **Table 1** showed, 45 cDNA libraries of ‘Ruidu Hongyu’ grape berries used for the RNA-seq, yielded a total of 481.02 G clean data. Also, the range of alignment efficiency was from 92.63% to 96.47%, and the raw reads ranged from 55124564 to 105458290 in JD3_3.

**Table 1:** Sequencing Data Statistics.

Sample	Raw_reads	Clean_reads	Clean_bases	Error (%)	Q20 (%)	Q30 (%)	GC (%)
B11	85205890	85205826	12.72	0.02	98.36	94.65	46.08
B12	70828280	70828238	10.58	0.02	98.03	93.65	46.31
B13	71209844	71209768	10.64	0.02	98.26	94.36	46.18
B21	76112350	76112298	11.38	0.02	98.19	94.16	46.38
B22	81824742	81824712	12.24	0.02	98.41	94.86	46.81
B23	65970108	65970048	9.87	0.02	98.48	95.04	46.66
B31	68628502	68628432	10.25	0.02	98.27	94.45	47.16
B32	62788950	62788916	9.38	0.02	98.22	94.3	47.28
B33	67081954	67042060	10.01	0.02	98.3	94.72	47.43
BB11	75942732	75942656	11.34	0.02	98.46	94.93	45.94
BB12	60226030	60225968	8.99	0.02	98.54	95.17	46.2
BB13	64036836	64036766	9.57	0.02	98.33	94.55	46.27
BB21	74304284	74304232	11.12	0.02	98.27	94.37	46.58
BB22	84485852	84485764	12.64	0.02	98.42	94.89	46.52
BB23	89641662	89641540	13.41	0.02	98.73	95.83	46.55
BB31	77559776	77559730	11.57	0.02	98.28	94.5	46.82
BB32	72400356	72400272	10.81	0.02	98.6	95.48	46.76
BB33	58309420	58309354	8.71	0.02	98.43	94.96	47.05
C11	75063212	75063172	11.22	0.02	98.34	94.54	46.16
C12	72163194	72163150	10.75	0.02	98.64	95.56	45.77
C13	78152558	78152470	11.69	0.02	98.72	95.79	46.7
C21	74826528	74785528	11.18	0.02	97.37	92.63	47.06
C22	76817800	76817744	11.48	0.02	98.35	94.65	46.84
C23	85672160	85672098	12.82	0.02	98.56	95.29	47.28
C31	67357674	67357594	10.07	0.02	98.31	94.54	46.9
C32	75191658	75191576	11.24	0.02	98.42	94.88	46.62
C33	62301866	62301812	9.31	0.02	98.35	94.62	46.69
J11	69506590	69506546	10.38	0.02	98.34	94.56	46.36
J12	70793104	70793036	10.58	0.02	98.25	94.2	46.82
J13	71361598	71361540	10.66	0.02	98.33	94.47	46.8
J21	64102882	64102816	9.56	0.02	98.44	94.92	47.24
J22	69342032	69341968	10.37	0.02	98.47	95.01	47.07
J23	55515572	55515504	8.29	0.02	98.66	95.59	46.86
J31	70277726	70277666	10.49	0.02	98.36	94.71	47.41
J32	65054898	65054852	9.72	0.02	98.29	94.49	46.7
J33	55124564	55124512	8.24	0.02	98.27	94.44	46.93
JD11	70448130	70408432	10.53	0.02	98.8	96.07	46.53
JD12	74537414	74494786	11.14	0.02	98.75	95.92	46.32
JD13	61383818	61349266	9.18	0.02	98.93	96.47	46.69
JD21	59637096	59637030	8.92	0.02	98.48	95.03	46.63
JD22	78744870	78744812	11.77	0.02	98.47	95	46.42
JD23	72776262	72776224	10.88	0.02	98.37	94.7	46.8
JD31	55151886	55151842	8.24	0.02	98.47	95.04	46.52
JD32	75802018	75801966	11.33	0.02	98.38	94.76	46.65
JD33	1.05E+08	1.05E+08	15.75	0.02	98.27	94.87	47.17

**Figure 3:**
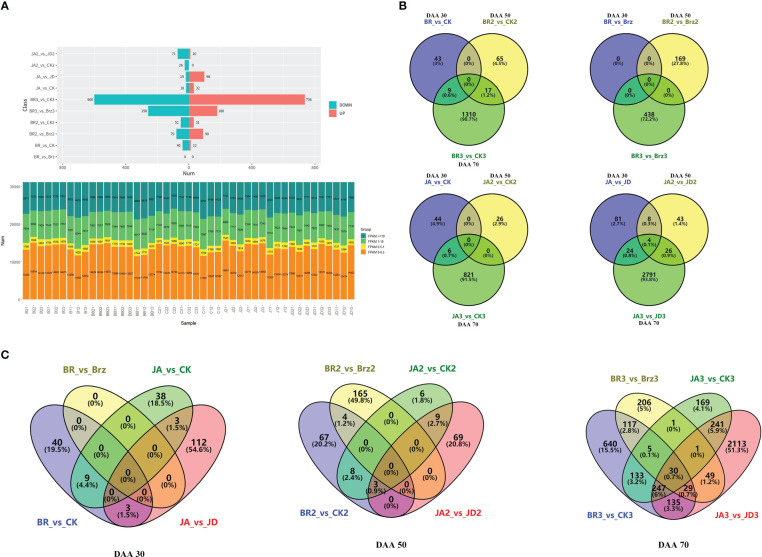
Summary of the number of DEGs identified by RNA-seq analysis under BR/JA/BB/JD treatments vs. CK groups. **(A)** Up-regulated DEGs and down-regulated DEGs in each comparison group. **(B, C)** Up-regulated DEGs and down-regulated DEGs in each comparison group are presented by Venn diagrams.

There was a total of 81 DEGs in JA_2 vs. JD_2, a total of 26 DEGs in JA_2 vs. CK_2, a total of 117 DEGs in JA_1 vs. JD_1, a total of 50 DEGs in JA_1 vs. CK_1, a total of 2845 DEGs in JA_3 vs. JD_3, a total of 827 DEGs in JA_3 vs. CK_3, a total of 1336 DEGs in BR_3 vs. CK_3, a total of 438 DEGs in BR_3 vs. Brz_3, a total of 82 DEGs in BR_2 vs. CK_2, a total of 169 DEGs in BR_2 vs. Brz_2, a total of 52 DEGs in BR_1 vs. CK1, a total of 0 DEGs in BR_1 vs. Brz_1. Among them, we found there were more down-regulated DEGs than up-regulated DEGs, except for JA_1 vs. JD_1 (19 down-regulated, and 98 up-regulated), BR_3 vs. CK_3 (600 down-regulated, and 736 up-regulated) and BR_2 vs. Brz_2 (79 down-regulated, and 90 up-regulated) groups (**Figure 3A**). As shown in **Figures 3B, C**, the Venn diagram further explained that at the grape ripening stage (DAA 70), the EBR and JA treatment groups had more DEGs than the other groups.

**Figure 4:**
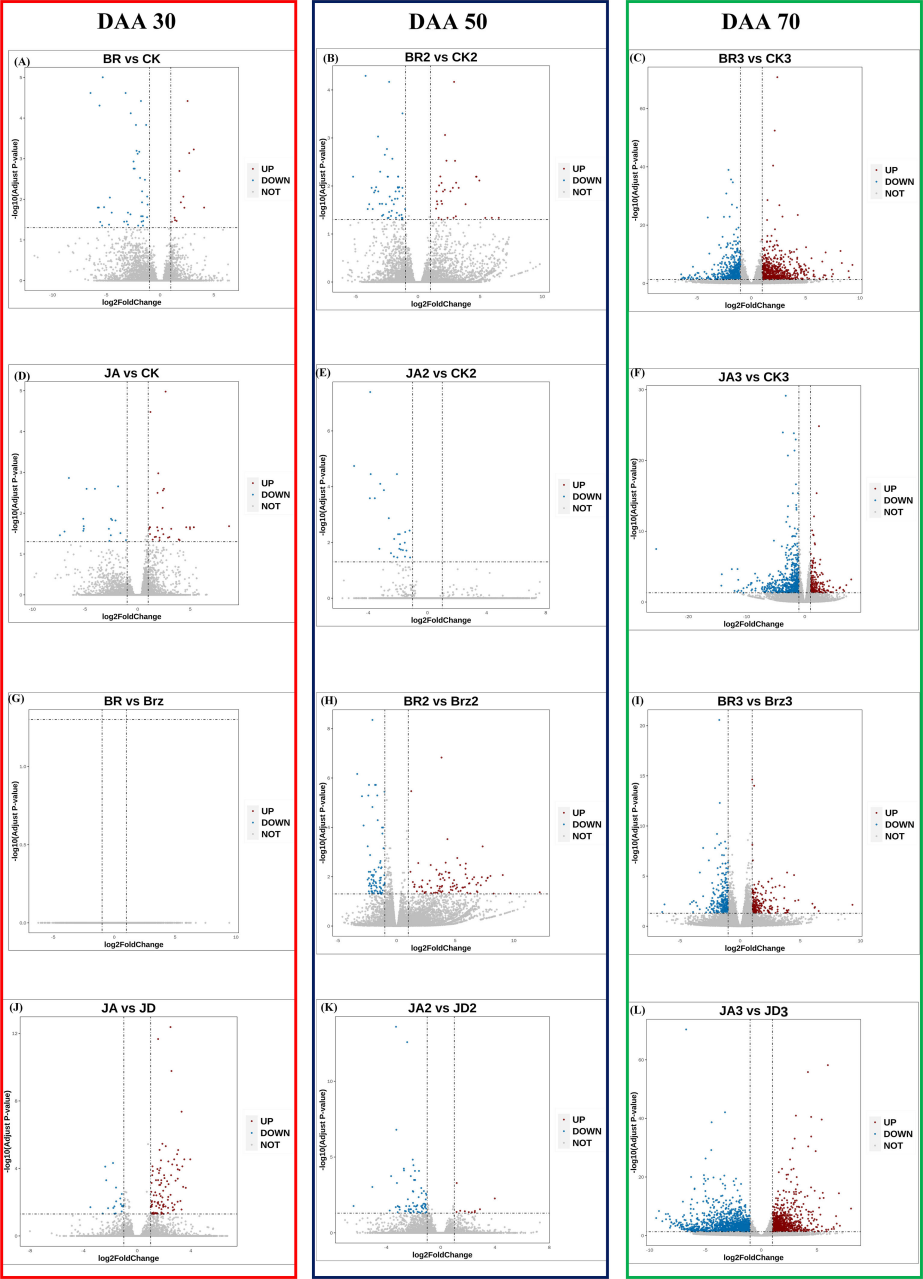
Differential expression volcano map of BR/JA/BB/JD vs. CK at three stages (DAA 30, 50, 70). Each point in the differential expression volcano map represents a gene. The blue dotsrepresented down-regulated DEGs, the red dots represented up-regulated DEGs, and the gray dots represented non-differentially expressed genes. **(A, D, G, J)** Aromatic series values at DAA 30. **(B, E, H, K)** Aromatic series values at DAA 50. **(C, F, I, L)** Aromatic series values at DAA 70.

The volcano plots could show the distribution of gene expression level differences between the two groups of samples. There were few up-regulated and down-regulated DEGs observed at DAA30 (**Figures 4A, D, G, J**), whereas DAA 50 (**Figures 4B, E, H, K**) and DAA 70 (**Figures 4C, F, I, L**) had more up-regulated. Moreover, the number of down-regulated DEGs was redundant with that of up-regulated DEGs. Consistent with the results of previous results (**Figure 3A**), there were also more up-regulated DEGs in the groups of JA_1 vs. JD_1, BR_3 vs. CK_3, and BR_2 vs. Brz_2.

**Figure 5:**
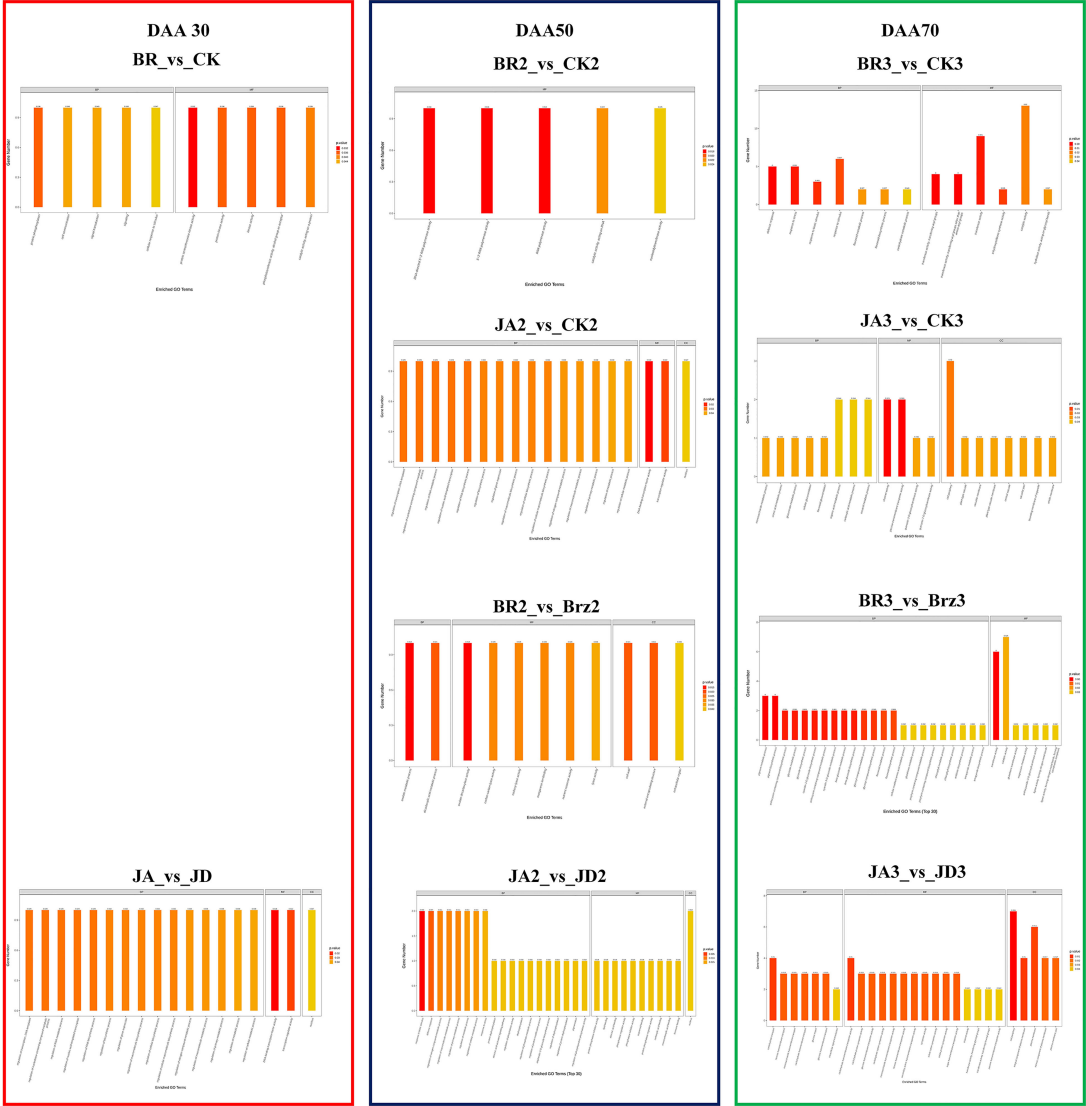
Gene ontology (GO) enrichment of DEGs among different comparison groups (BR/JA/BB/JD treatments vs. CK). The DEGs were enriched into three classifications of biological process (BP), molecular function (MF), and cellular components (CC).

The functional annotation of databases for DEGs may be better understood through GO functional enrichment analysis. In this study, GO enrichment was displayed in three main categories: biological process (BP), molecular function (MF), and cellular component (CC) (**Figure 5**). Specifically, more functional action was observed at late fruit ripening stages (DAA 70). The catalytic activity of DEGs was primarily enriched in the BR_3 vs. CK_3 and BR_3 vs. Brz_3 groups, whereas the cell periphery was mainly enhanced in the JA_3 vs. CK_3 and JA_1 vs. JD_1 groups. Moreover, the fact is that EBR and JA encouraged the accumulation of secondary metabolites in grapevine in the form of alcohols, aldehydes, and terpenes. Above results from the side confirmed that at the late fruit ripening stage, EBR and JA promoted a large amount of volatiles accumulation.

**Figure 6:**
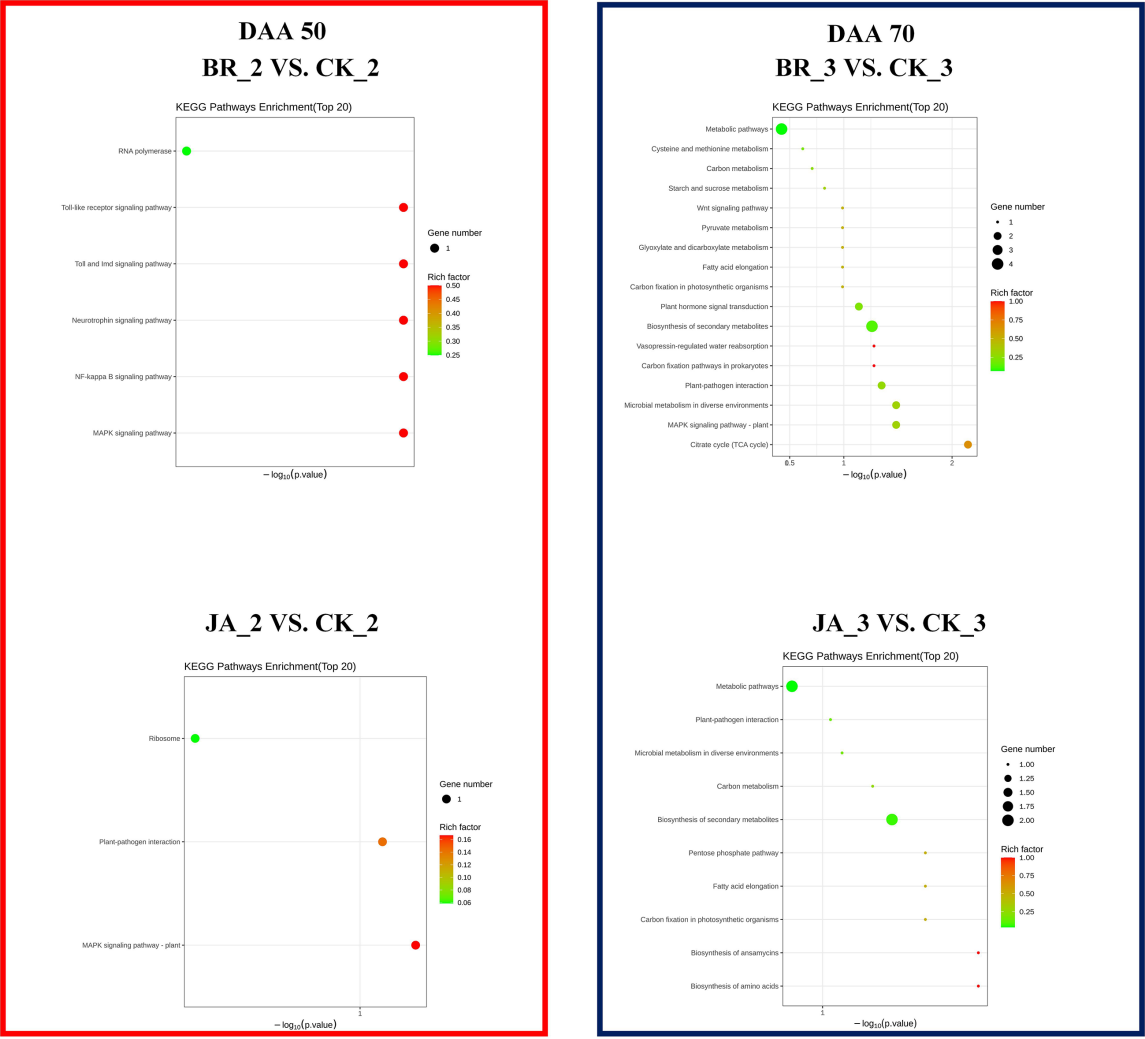
KEGG enrichment analysis of the DEGs among BR_2 vs. CK_2, BR_3 vs. CK_3, JA_2 vs. CK_2, JA_3 vs. CK_3 treatments.

Unlike GO enrichment analysis, KEGG not only has gene sets but also defines the complex interrelationship between related genes and metabolites. In accordance with **Figure 6**, the RNA polymerase process and metabolic pathway, plant hormone signal transduction, and biosynthesis of secondary metabolites were the main areas where DEGs were enriched in the BR_2 vs. CK_2 group and BR_3 vs. CK_3 group, respectively. Regarding JA_2 vs. CK_2 group, DEGs were mainly enriched in the ribosome pathway. In addition, there were found more DEGs that were enriched in metabolic pathways and secondary metabolite biosynthesis in the JA_3 vs. CK_3 group. These findings further demonstrated that EBR and JA could promote the accumulation of multiple secondary metabolites and the occurrence of multiple metabolic activities in grapevines. And both EBR and JA treatments might promote the metabolism of fatty acids or amino acids at the late stage of fruit ripening.

### DEGs involved in terpene, alcohol and aldehyde biosynthesis and metabolism

**Figure 7:**
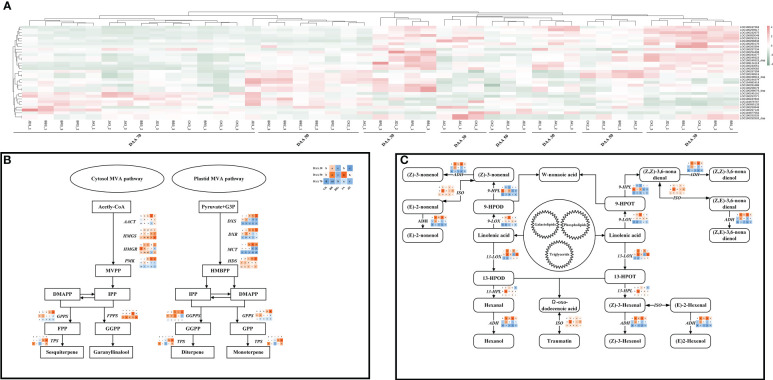
Heatmap and key gene expression profiles involved in the terpene/alcohol/aldehyde biosynthesis pathways. **(A)** Heat map for key genes involved in the biosynthesis of terpene compounds. **(B)** Key gene expression profiles involved in the terpene biosynthesis pathways. **(C)** Key gene expression profiles involved in the alcohol and aldehyde biosynthesis pathways.

In this study, exogenous EBR and JA had a significant impact on the biosynthesis of terpenes, alcohols, and aldehydes. To further understand the effect of exogenous EBR and JA on aroma compounds biosynthesis and metabolism-related genes at the transcription level, heat map analysis was carried out based on the results of the transcriptome analysis (**Figure 7A**). The information of specific key gene selected in this study was shown in **Table 2**. In this study, the pattern of gene expression by performing a K-means cluster analysis on the time series for genes related to terpene, alcohol, and aldehyde biosynthesis. There were two clusters formed, and cluster 1 was characterized by a significant increase in transcript levels at DAA 30 for both the EBR and JA treatments. Interestingly, terpene biosynthesis genes like *LOC100247263*, *LOC100259617*, *LOC100232975*, and *LOC100855049* were upregulated by BB and JD treatments, which also contributed to terpene accumulation at DAA 30. Moreover, BB and JD treatments also contributed to terpene accumulation at DAA 30 and up-regulated the expression of genes related to terpene biosynthesis, including *LOC100247263*, *LOC100259617*, *LOC100232975*, and *LOC100855049*. When exposed to EBR and JA at DAA 70, the terpene biosynthesis genes in cluster 2 (*LOC100247834*, *LOC104878767*, and *LOC100855133*) showed a noticeably increased transcript level. These genes were also expressed more strongly in the BB and JD groups than in the CK group at DAA 70.

**Table 2:** The information of key genes in aroma volatiles (terpenes, alcohol, aldehyde) biosynthesis pathways.

Gene id	Symbol	Description
gene-LOC100244379	XP_002282434.1	PREDICTED: hydroxymethylglutaryl-CoA synthase [Vitis vinifera]|CBI34763.3 unnamed protein product, partial [Vitis vinifera]
gene-LOC100250849	RVW44176.1	Hydroxymethylglutaryl-CoA synthase [Vitis vinifera]
gene-LOC104882418	XP_010663892.1	PREDICTED: hydroxymethylglutaryl-CoA synthase [Vitis vinifera]
gene-LOC100254498	RVW44176.1	Hydroxymethylglutaryl-CoA synthase [Vitis vinifera]
gene-LOC100243051	XP_002265638.1	PREDICTED: 3-hydroxy-3-methylglutaryl-coenzyme A reductase 1 [Vitis vinifera]
gene-LOC100265082	RVW50943.1	3-hydroxy-3-methylglutaryl-coenzyme A reductase 1 [Vitis vinifera]
gene-LOC100245191	XP_002283183.2	PREDICTED: 3-hydroxy-3-methylglutaryl-coenzyme A reductase [Vitis vinifera]
gene-LOC100251686	RVW94263.1	Diphosphomevalonate decarboxylase MVD2, peroxisomal [Vitis vinifera]
gene-LOC100232975	RVX14814.1	Farnesyl pyrophosphate synthase 1 [Vitis vinifera]
gene-LOC100253115	XP_002273133.1	PREDICTED: geranylgeranyl diphosphate reductase, chloroplastic [Vitis vinifera]
gene-LOC100243277	XP_002284906.1	PREDICTED: geranylgeranyl diphosphate reductase, chloroplastic [Vitis vinifera]
gene-LOC104877552	XP_019072635.1	PREDICTED: sesquiterpene synthase-like isoform X1 [Vitis vinifera]
gene-LOC100260170	XP_002275106.1	PREDICTED: probable terpene synthase 9 [Vitis vinifera]|RVW72494.1 putative terpene synthase 9 [Vitis vinifera]
gene-LOC100267145	NP_001268063.1	uncharacterized protein LOC100267145 [Vitis vinifera]|ADR74196.1 terpene synthase [Vitis vinifera]
gene-LOC100268079	XP_002266925.1	PREDICTED: probable 1-deoxy-D-xylulose-5-phosphate synthase 2, chloroplastic [Vitis vinifera]|CBI40852.3 unnamed protein product, partial [Vitis vinifera]
gene-LOC100252520	RVX19907.1	1-deoxy-D-xylulose-5-phosphate synthase 1, chloroplastic [Vitis vinifera]
gene-LOC100249323	RVX21930.1	putative 1-deoxy-D-xylulose-5-phosphate synthase, chloroplastic [Vitis vinifera]
gene-LOC100248516	PPS01508.1	hypothetical protein GOBAR_AA19160 [Gossypium barbadense]
gene-LOC100248516	PPS01508.1	hypothetical protein GOBAR_AA19160 [Gossypium barbadense]
gene-LOC100249323	RVX21930.1	putative 1-deoxy-D-xylulose-5-phosphate synthase, chloroplastic [Vitis vinifera]
gene-LOC100268079	XP_002266925.1	PREDICTED: probable 1-deoxy-D-xylulose-5-phosphate synthase 2, chloroplastic [Vitis vinifera]|CBI40852.3 unnamed protein product, partial [Vitis vinifera]
gene-LOC100247834	RVW95950.1	hypothetical protein CK203_027769 [Vitis vinifera]
gene-LOC100263394	CBI17808.3	unnamed protein product, partial [Vitis vinifera]
gene-LOC100252520	RVX19907.1	1-deoxy-D-xylulose-5-phosphate synthase 1, chloroplastic [Vitis vinifera]
gene-LOC109124119	–	–
gene-LOC100855049	–	–
gene-LOC100247263	RVX05912.1	2-C-methyl-D-erythritol 4-phosphate cytidylyltransferase, chloroplastic [Vitis vinifera]
gene-LOC100257071	XP_002285130.1	PREDICTED: 4-hydroxy-3-methylbut-2-en-1-yl diphosphate synthase (ferredoxin), chloroplastic [Vitis vinifera]|CBI16309.3 unnamed protein product, partial [Vitis vinifera]
gene-LOC104878767	XP_010647702.1	PREDICTED: heterodimeric geranylgeranyl pyrophosphate synthase small subunit, chloroplastic-like [Vitis vinifera]
gene-LOC100259617	XP_010649718.1	PREDICTED: heterodimeric geranylgeranyl pyrophosphate synthase large subunit 1, chloroplastic [Vitis vinifera]|RVW55636.1 Heterodimeric geranylgeranyl pyrophosphate synthase large subunit 1, chloroplastic [Vitis vinifera]
gene-LOC100266842	XP_002273789.1	PREDICTED: geranylgeranyl pyrophosphate synthase, chloroplastic [Vitis vinifera]|RVW73271.1 Geranylgeranyl pyrophosphate synthase, chloroplastic [Vitis vinifera]
gene-LOC100855133	XP_003631563.1	PREDICTED: heterodimeric geranylgeranyl pyrophosphate synthase small subunit, chloroplastic [Vitis vinifera]
gene-LOC100257359	XP_002283364.1	PREDICTED: geranylgeranyl pyrophosphate synthase, chloroplastic [Vitis vinifera]
gene-LOC100257234	XP_002278023.1	PREDICTED: heterodimeric geranylgeranyl pyrophosphate synthase small subunit, chloroplastic [Vitis vinifera]|RVW99607.1 Geranylgeranyl pyrophosphate synthase, chloroplastic [Vitis vinifera]
gene-LOC100244379	XP_002282434.1	PREDICTED: hydroxymethylglutaryl-CoA synthase [Vitis vinifera]|CBI34763.3 unnamed protein product, partial [Vitis vinifera]
gene-LOC100250849	RVW44176.1	Hydroxymethylglutaryl-CoA synthase [Vitis vinifera]
gene-LOC104882418	XP_010663892.1	PREDICTED: hydroxymethylglutaryl-CoA synthase [Vitis vinifera]
gene-LOC100254498	RVW44176.1	Hydroxymethylglutaryl-CoA synthase [Vitis vinifera]
gene-LOC100243051	XP_002265638.1	PREDICTED: 3-hydroxy-3-methylglutaryl-coenzyme A reductase 1 [Vitis vinifera]
gene-LOC100265082	RVW50943.1	3-hydroxy-3-methylglutaryl-coenzyme A reductase 1 [Vitis vinifera]
gene-LOC100245191	XP_002283183.2	PREDICTED: 3-hydroxy-3-methylglutaryl-coenzyme A reductase [Vitis vinifera]
gene-LOC100251686	RVW94263.1	Diphosphomevalonate decarboxylase MVD2, peroxisomal [Vitis vinifera]
gene-LOC100232975	RVX14814.1	Farnesyl pyrophosphate synthase 1 [Vitis vinifera]

The qRT-PCR analysis was conducted to further confirm the regulation mechanism of EBR and JA on aroma substance biosynthesis. The expression of *AACT*, *HMGS* (responsible for MVPP accumulation), *FPPS* (responsible for geranyl linalool accumulation), and *TPS* (responsible for sesquiterpene accumulation) genes in the MVA pathway (cytosol) were up-regulated under EBR and JA treatments at DAA 30 and DAA 50 (**Figure 7B**). Moreover, the higher expression of *DXS*, *DXR*, *MCT*, *HDS* (responsible for HMBPP accumulation), *GGPPS*, and *TPS* (responsible for diterpene accumulation) were also found in the MVA pathway (plastid) under EBR and JA treatments at DAA 30. It is noteworthy that BB and JD treatments down-regulated the expression of these genes throughout the study. There was a total of six genes whose expressions were found in relation to the patterns of alcohol and aldehyde biosynthesis and metabolism shown in **Figure 7C**. According to the findings, EBR and JA treatments were responsible to increase the expression of *ADH*, *9-LOX*, and *13-HPL* at DAA 30 and DAA 50, whereas the expression of *9-HPL* and *13-LOX* was found higher at DAA 70. In addition, we found EBR and JA treatments promoted ISO expression at all sampling stages. Overall, BB and JD application consistently decreased the expressions of these genes.

### DEGs involved in BR and JA biosynthesis, metabolism and signaling

**Figure 8:**
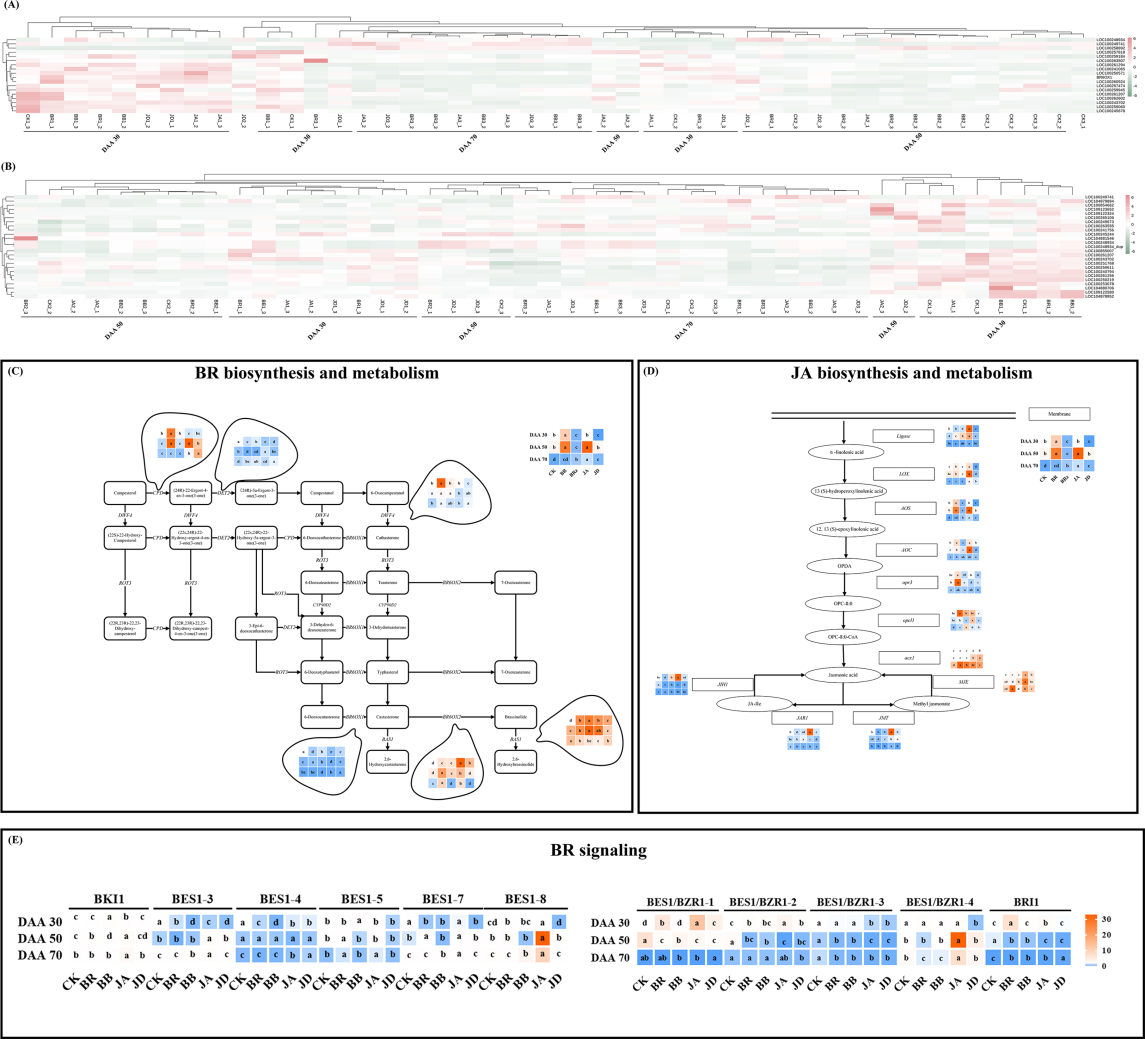
Heatmap and key gene expression profiles involved in the BR/JA biosynthesis, metabolism/signaling pathways. **(A)** Heat map for key genes involved in the biosynthesis of endogenous BR. **(B)** Heat map for key genes involved in the biosynthesis of endogenous JA. **(C)** Key gene expression profiles involved in the BR biosynthesis and metabolism pathways. **(D)** Key gene expression profiles involved in JA biosynthesis and metabolism pathways. **(E)** Key transcription factors expression profiles involved in BR signaling pathways.

Using exogenous EBR and JA application must influence endogenous BR and JA biosynthesis. In this study, the gene expression patterns of BR and JA biosynthesis and metabolism were investigated in the basis of K-means value. The specific information of key gene selected in this study was shown in **Table 3**. As **Figure 8A** displayed, there were also 2 clusters generated in BR biosynthesis pathway, cluster 2 was identified by an increase in transcript (*LOC100257818*, *LOC100259184*, *LOC100263507*, *LOC100263507*, *LOC100261294*, *LOC100241065*, *LOC100250571*, *BR6OX1*, *LOC100260924*, *LOC100257474*, *LOC100259945*, *LOC100261207*, *LOC100262602*, *LOC100243702*, *LOC100259060*, *LOC100245678*) under both EBR and JA treatments at DAA 30. Differently, cluster 1 was determined by an increase in transcript (*LOC100248934*, *LOC100249741*, *LOC100258692*) under EBR and JA treatments at DAA 70. Moreover, we found treatments containing signaling inhibitors (BB and JD) could effectively inhibit these gene expressions at all sampling stages. As **Figure 8B** displayed, there were also 2 clusters generated in JA biosynthesis pathway. Cluster 1, which contained *LOC100249741*, *LOC104879884*, *LOC100854662*, was identified by an increase of transcript in EBR and JA treatment groups at DAA 70. Furthermore, cluster 2, which included *LOC100253078*, *LOC104880706*, *LOC109122580*, *LOC104878952*, was defined by an increase of transcript in EBR treatment group at DAA 30.

**Table 3:** The information of key genes in BR biosynthesis, metabolism, and signaling pathways.

Gene id	Symbol	Description
gene-LOC100261207	XP_002281730.1	PREDICTED: serine/threonine-protein kinase BRI1-like 2 [Vitis vinifera]
gene-LOC100248934	XP_010656506.1	PREDICTED: serine/threonine-protein kinase BRI1-like 1 isoform X2 [Vitis vinifera]
gene-LOC100243702	XP_002284646.1	PREDICTED: BRI1 kinase inhibitor 1 [Vitis vinifera]
gene-LOC100249741	XP_002265525.3	PREDICTED: receptor-like protein kinase BRI1-like 3 [Vitis vinifera]|RVW60694.1 Receptor-like protein kinase BRI1-like 3 [Vitis vinifera]
gene-LOC100263555	XP_002267082.1	PREDICTED: BES1/BZR1 homolog protein 4 isoform X1 [Vitis vinifera]|XP_019078200.1 PREDICTED: BES1/BZR1 homolog protein 4 isoform X2 [Vitis vinifera]
gene-LOC100854662	XP_003632998.2	PREDICTED: protein BZR1 homolog 3 [Vitis vinifera]
gene-LOC100259611	XP_002277666.1	PREDICTED: calnexin homolog [Vitis vinifera]
gene-LOC100261256	CAN64359.1	hypothetical protein VITISV_041427, partial [Vitis vinifera]
gene-LOC100241756	RVX03915.1	BES1/BZR1-like protein 4 [Vitis vinifera]
gene-LOC100249673	XP_002273547.1	PREDICTED: BES1/BZR1 homolog protein 2 [Vitis vinifera]|RVW23059.1 BES1/BZR1-like protein 2 [Vitis vinifera]|RVW73998.1 BES1/BZR1-like protein 2 [Vitis vinifera]
gene-LOC109123652	RVW69911.1	Serine/threonine-protein phosphatase 7 long form-like [Vitis vinifera]
gene-LOC100245244	RVW79881.1	Serine/threonine-protein phosphatase PP-X isozyme 2 [Vitis vinifera]
gene-LOC100265106	RVW40108.1	Serine/threonine-protein phosphatase 7 long form-like [Vitis vinifera]
gene-LOC100855007	XP_010656947.1	PREDICTED: serine/threonine-protein phosphatase 7 long form homolog isoform X1 [Vitis vinifera]|XP_010656949.1 PREDICTED: serine/threonine-protein phosphatase 7 long form homolog isoform X1 [Vitis vinifera]
gene-LOC100240794	XP_010656947.1	Serine/threonine-protein phosphatase PP1 [Vitis vinifera]
gene-LOC109122580	RVW52005.1	Serine/threonine-protein phosphatase 7 long form-like [Vitis vinifera]
gene-LOC109122324	RVW83178.1	Serine/threonine-protein phosphatase 7 long form-like [Vitis vinifera]
gene-LOC104880706	RVW38110.1	Serine/threonine-protein phosphatase 7 long form-like [Vitis vinifera]
gene-LOC104881546	XP_010645203.2	PREDICTED: serine/threonine-protein phosphatase 7 long form homolog [Vitis vinifera]
gene-LOC100251768	XP_002263740.1	PREDICTED: serine/threonine-protein phosphatase 7 isoform X1 [Vitis vinifera]
gene-LOC100250219	XP_010650977.1	PREDICTED: serine/threonine-protein phosphatase PP1 [Vitis vinifera]|XP_010650978.1 PREDICTED: serine/threonine-protein phosphatase PP1 [Vitis vinifera]|CBI16052.3 unnamed protein product, partial [Vitis vinifera]
gene-LOC104879884	RVW65182.1	Serine/threonine-protein phosphatase 7 long form-like [Vitis vinifera]
gene-LOC100253078	RVW69314.1	Serine/threonine-protein phosphatase 7 long form-like [Vitis vinifera]
gene-LOC104878952	RVW13658.1	Serine/threonine-protein phosphatase 7 long form-like [Vitis vinifera]
gene-LOC100248934	XP_010656506.1	PREDICTED: serine/threonine-protein kinase BRI1-like 1 isoform X2 [Vitis vinifera]
gene-LOC100257818	XP_002273613.1	PREDICTED: cytochrome P450 85A isoform X1 [Vitis vinifera]|CBI27310.3 unnamed protein product, partial [Vitis vinifera]
gene-BR6OX1	RVW98059.1	Cytochrome P450 85A1 [Vitis vinifera]
gene-LOC100248934	XP_010656506.1	PREDICTED: serine/threonine-protein kinase BRI1-like 1 isoform X2 [Vitis vinifera]
gene-LOC100261207	XP_002281730.1	PREDICTED: serine/threonine-protein kinase BRI1-like 2 [Vitis vinifera]
gene-LOC100243702	XP_002284646.1	PREDICTED: BRI1 kinase inhibitor 1 [Vitis vinifera]
gene-LOC100249741	XP_002265525.3	PREDICTED: receptor-like protein kinase BRI1-like 3 [Vitis vinifera]|RVW60694.1 Receptor-like protein kinase BRI1-like 3 [Vitis vinifera]
gene-LOC100261294	XP_002277900.1	PREDICTED: steroid 5-alpha-reductase DET2 [Vitis vinifera]|CBI30434.3 unnamed protein product, partial [Vitis vinifera]|RVW15971.1 Steroid 5-alpha-reductase DET2 [Vitis vinifera]
gene-LOC100259184	XP_002275776.1	PREDICTED: steroid 5-alpha-reductase DET2 [Vitis vinifera]|XP_019073162.1 PREDICTED: steroid 5-alpha-reductase DET2 [Vitis vinifera]
gene-LOC100258692	RVW81366.1	Cytochrome P450 90A1 [Vitis vinifera]
gene-LOC100259060	RVW51739.1	3-epi-6-deoxocathasterone 23-monooxygenase [Vitis vinifera]
gene-LOC100259945	XP_010654520.1	PREDICTED: 3-epi-6-deoxocathasterone 23-monooxygenase [Vitis vinifera]|CBI35940.3 unnamed protein product, partial [Vitis vinifera]
gene-LOC100262602	RVX15113.1	Cytochrome P450 709B2 [Vitis vinifera]
gene-LOC100257474	XP_002281661.1	PREDICTED: cytochrome P450 734A1 [Vitis vinifera]|RVW50410.1 Cytochrome P450 734A1 [Vitis vinifera]
gene-LOC100263507	XP_002281365.1	PREDICTED: cytochrome P450 734A1 [Vitis vinifera]
gene-LOC100250571	RVX15111.1	Cytochrome P450 734A1 [Vitis vinifera]
gene-LOC100245678	XP_002285021.2	PREDICTED: cytochrome P450 734A1 [Vitis vinifera]
gene-LOC100260924	–	–
gene-LOC100241065	CBI17531.3	unnamed protein product, partial [Vitis vinifera]

In addition, qRT-PCR was carried out to further verify the effect of EBR and JA on BR/JA biosynthesis and metabolism at transcription level. There are six genes involved in BR biosynthesis and metabolism, and eleven transcription factors related to BR signaling. We found *DET2* and *DWF4* (upstream genes for BR biosynthesis), *BR6OX1* and *BR6OX2* (downstream genes for BR biosynthesis), were up-regulated under EBR and JA treatments at DAA 70. *CPD* (upstream gene for BR biosynthesis) and *BAS1* (key gene for BR metabolism) were up-regulated under EBR and JA treatments at DAA 50 (**Figure 8C**). Moreover, as far as BR signaling is concerned, BKI1/BES1-3/BES1-5/BES1-7/BES1-8 were up-regulated under EBR and JA treatments at DAA 70, while BES1/BZR1-1/2/4, as well as, BRI1 was up-regulated under EBR and JA treatments at DAA 30 (**Figure 8E**). In addition, we found BB and JD treatments inhibited most of the above gene expressions, especially at early fruit ripening stages (DAA 30).

In this study, we also quantified the expression patterns of genes for JA biosynthesis and metabolism. The results illustrated that *Ligase*, *LOX*, *AOS*, *AOC*, *opcl1*, *acx1* (genes for JA biosynthesis) were expressed at a higher level in EBR and JA groups at DAA 50. At DAA 30, the expression levels of *opr3* and *MJE*, which contributed to JA biosynthesis, also increased under EBR and JA application. Moreover, exogenous EBR down-regulated the expressions of *JAR1* and *JMT* (genes for JA metabolism) at DAA 30 and DAA 70, while exogenous JA significantly up-regulated them at DAA 30 (**Figure 8D**). More consistently, the above gene expression was inhibited by BB and JD treatments, especially at late fruit ripening stages (DAA 50 or DAA 70).

## Discussion

### Exogenous EBR and JA promoted biochemical properties of grape berries

Exogenous phytohormones are used to improve the berries’ quality while the grapevine is growing and developing. According to prior research, EBR could significantly increase grape berry size and sugar content as well as improved grape color and flavor formation (Li et al., 2022; Lv et al., 2022a). Similarly, JA could also contribute to berry expansion, sugar accumulation, and anthocyanins formation (Yu et al., 2020; Gao et al., 2022; Wang et al., 2022). In agreement with the results of previous research (Li et al., 2022), this study further verified the results that EBR and JA also promoted the biochemical properties of ‘Ruidu Hongyu’ grapevine. We investigated more closely than in the previous study to assess the effect of EBR and JA on the biosynthesis of grape berry free-form aroma volatiles. The results revealed that exogenous EBR and JA had a significant promoting effect on free-form aroma substances (aldehydes, hotrienol, terpineol, nerol, geraniol, linalool, and citral) at late fruit ripening stages (DAA 70). Moreover, it was found that exogenous EBR and JA were primarily responsible for the production of rose-aroma characteristics of grape berries. The aforementioned findings supported earlier research that showed exogenous EBR and JA promoted aroma volatilization and flavor improvement (Xi et al., 2013; Li et al., 2022; Wang et al., 2022).

### Transcriptome sequencing revealed exogenous EBR and JA accelerated aroma substances accumulation of grape berries

Sequencing quality revealed sequencing depth and repeatability (Barbitoff et al., 2020). In this study, the amount of high-quality data of the sequenced sample was more than 481.02 G worth, the Q30 base distribution was more than 92%, clean reads rate mapped to the grapevine genome was more than 90%. In all, we concluded that the quality and depth of the sequenced samples were high. Moreover, the outstanding repeatability among the samples was illustrated by the correlation coefficient (greater than 0.99). During the whole development of grapevine, there was a total of 3043 DEGs in JA vs. JD, a total of 903 DEGs in JA vs. CK, a total of 1470 DEGs in BR vs. CK, and a total of 607 DEGs in BR vs. Brz. Also, we found that only BR vs. CK and JA vs. CK groups at DAA 70 had more DEGs. In addition, there were more DEGs were down-regulated in JA_2 vs. JD_2, JA_2 vs. CK_2, BR_3 vs. Brz_3, BR_2 vs. CK2, BR vs. CK groups. The volcano plots further intuitively corroborated our conclusion.

GO enrichment analysis revealed the process of activities to which multiple DEGs responded (Yan et al., 2019; Zhu et al., 2019). Previous researchers’ findings suggested that EBR and JA promoted the accumulation of several terpenes and aldehydes (Li et al., 2021; Liu et al., 2022). The formation of secondary metabolites and catalytic activity were the main areas of enrichment for DEGs in comparison groups, and a similar result could be obtained by combining transcriptome data analysis (GO enrichment analysis). In the process of bioinformatics analysis, KEGG pathway enrichment analysis is often applied to the functional annotation of differentially expressed genes. Thus, the related functions and action pathways of differentially expressed genes were constructed (Yang et al., 2018; Jie et al., 2020). In this study, multiple secondary metabolites formation and the occurrence of various metabolic activities in grapevines were affected by exogenous EBR and JA. These further authenticated that the increase of terpenes and aldehydes contents might be regulated by exogenous EBR and JA signals. The outcomes were more consistent with previous findings that the exogenous EBR and JA were involved in regulating the biosynthesis of several metabolites, including monosaccharides, anthocyanins, fatty acids, amino acids, and so on, with significantly higher levels than the CK group (Mao et al., 2017; Vergara et al., 2018; Nakajima et al., 2021; Chen et al., 2022; Li et al., 2022; Lv et al., 2022a). Results of this study provided reference information for verifying the interaction of EBR and JA in regulating aroma substances biosynthesis. Therefore, DEGs identified in this study were worthy of further studies on the function of genes interactions to mediate various life activities in grapevine.

### Exogenous EBR and JA up-regulated the expressions of key genes related to aroma substances biosynthesis

EBR was reported as an effective phytohormone that had a significant impact on the aroma substances by up-regulating the gene expressions responsible for terpenes biosynthesis (*VvHMGR*) (Zheng et al., 2020). Moreover, the scientist explained that amino acid volatilization was accelerated by up-regulating the expression of the genes involved in amino acid biosynthesis (Lv et al., 2022b). Regarding JA, it also increased the genes expressions of *PgIPPI* and *PgFPS*, which promoted the accumulation of terpenes such as geraniolene, limonene, panasinsene, elemene, farnesane, alpha carophyllene, beta carophyllene, germacrene, bulsenol, citronellal, and falcrinol (Balusamy et al., 2015). Furtherly, to better understand how EBR and JA control the enrichment of aroma metabolites, qRT-PCR assays were used to measure the expression of key genes involved in the biosynthesis and metabolism of terpenes, alcohol, and aldehydes. In the early stage, most genes related to terpenes and aldehyde biosynthesis and metabolism could be divided into two clusters through transcriptome sequencing. At DAA 30 and 70, EBR and JA increase the expressions of most key genes involved in aroma substances biosynthesis. Through qRT-PCR, we further confirmed that EBR and JA had a facilitation effect on the expression of terpene and aldehyde biosynthetic genes. Genes involved in the MVA pathway (*AACT*, *HMGS*, *FPPS*) were up-regulated under both EBR and JA treatments at late fruit ripening stages (DAA 50). In the MVA pathway, both EBR and JA treatments increased the expression of genes such as *DXS*, *DXR*, *MCT*, *HDS*, *FPPS*, and *GGPPS* at the start of fruit ripening (DAA 30). The *ISO*, *9-LOX*, and *13-HPL* genes responsible for the formation of alcohol and aldehydes were also expressed at a higher level at DAA 50 in response to EBR and JA treatments. The relative expression levels were consistent with the overall trend of deep sequencing data. Further, the validity of the RNA Seq gene expression profiles was confirmed by qRT-PCR tests. Therefore, we concluded exogenous EBR and JA promoted the accumulation of aroma substances at late stages of fruit ripening. Moreover, the expression of most structural genes related to the biosynthesis of terpenes, alcohol and aldehydes were up-regulated. Previous studies illustrated that 100 μmol L^-1^ of exogenous BR promoted HMGR activity at veraison stages of five-year-old ‘Kyoho’ grapevine (Zheng et al., 2020) while 10 mM application of MeJA significantly up-regulated *DXS*, *HMGCR*, *TPS14* in the monoterpene biosynthesis pathway in grapevine (Li et al., 2020). Interestingly, we found that EBR and JA signaling inhibitors effectively reversed the gain effect of EBR and JA on the accumulation of aroma volatiles in grape berries. Therefore, we inferred that exogenous EBR and JA might accelerate the accumulation of aroma volatiles and grape flavor by enhancing the expression of several transcription factors, which were related to signal transduction of terpenes and other aroma compounds.

### Exogenous EBR and JA up-regulated the expressions of key genes related to BR/JA biosynthesis, metabolism and signaling

The application of exogenous phytohormones causes changes in the content of endogenous phytohormones, as well as the enhancement of phytohormones signal transduction (Fahad et al., 2015). It had been reported that EBR treatment up-regulated the expression of BR biosynthesis-related gene expressions (*CYP85A1*, *CYP85A3*). Concurrently, the expressions of BR signaling receptor proteins (BZR1-1D) were enhanced (Liu et al., 2014). In addition, exogenous JA is also responsible for boosting the accumulation of endogenous JA due to the up-regulated expression of JA synthetic structural genes and JA signaling receptors (Qiu et al., 2020). On the basis of transcriptome data, we concluded that exogenous EBR and JA significantly contribute to the rise in endogenous BR and JA contents at the late stage of berry ripening. Furthermore, qRT-PCR demonstrated that EBR and JA treatments at DAA 70 increased the expression of two key genes (*BR6OX1* and *BR6OX2*) that control BR biosynthesis. Six essential genes for JA biosynthesis, including *ligase*, *LOX*, *AOS*, *AOC*, *opcl1* and *acx1*, were expressed at higher levels in the EBR and JA groups at DAA 50. Moreover, BKI1, BES1-3, BES1-5, BES1-7, and BES1-8, which were recognized as key transcription factors for BR signaling, were up-regulated under EBR and JA treatments at DAA 70. The relative expression levels were consistent with the overall trend of deep sequencing data, as well. Previous studies illustrated that 0.40 mg L^-1^ of 24-Epibrassinolide treatment altered endogenous BR accumulation in ‘Cabernet Sauvignon’ grapevine. The expressions of BRs biosynthetic enzymes (*VvBR6OX1* and *VvDWF1*) were down-regulated, while the expression of BR receptor gene (VvBRI1) was up-regulated (Xu et al., 2015). Moreover, 10 mM of MeJA significantly up-regulated expression levels of *LOX2S*, *AOS*, *OPR*, and *JMT* in the JA biosynthesis pathway (Li et al., 2020). These findings were accordant with the results obtained in this study. These findings led us to the conclusion that exogenous EBR and JA enhanced the expression of key genes involved in BR/JA biosynthesis, metabolism, and signaling, resulting in the enrichment of endogenous BR and JA in the grapevine. Furthermore, we found that inhibitors of EBR and JA signaling efficiently reversed the gain effect of EBR and JA on the biosynthesis of endogenous BR and JA in grape berries. We can summarize from this that EBR and JA may influence BR and JA signal transduction in a manner that causes endogenous BR and JA levels to rise at the late stages of berry ripening.

## Conclusion

Overall, exogenous EBR and JA application not only promoted improvement of external qualities (berries expansion and coloration) but also significantly increased the content of free-form aroma substances (terpenes, alcohol, aldehyde). The mechanism by which EBR and JA control the accumulation of aroma volatiles has been better understood through transcriptome sequencing. A total of 3043, 903, 1470 and 607 DEGs were identified in JA vs. JD, JA vs. CK, BR vs. CK, BR vs. Brz, respectively. There were more DEGs under both EBR and JA treatments at late fruit ripening stages than at early fruit ripening stages. Moreover, qRT-PCR revealed that the expression of various genes involved in the biosynthesis of terpenes, alcohol, and aldehydes was increased in response to the EBR and JA treatments. Most of the genes involved in BR and JA biosynthesis and signaling were up-regulated concurrently. In conclusion, these findings provide new insight for the investigation of the DEGs (responsible for aroma substances enrichment) under EBR and JA treatments. Further investigation is required into the molecular mechanisms affecting the interaction of phytohormones in improving grape flavor.

## Data availability statement

The datasets presented in this study can be found in online repositories. The names of the repository/repositories and accession number(s) can be found in the article/**Supplementary Material**.

## Author contributions

JL carried out all experiments, data analyses, draft composed and figures drawn. ZW and YQ ground samples. JH and YZ provided all experimental materials. CM, SJ, CZ reviewed the manuscript. LW and HJ designed the experiments and provided ideas. SW supervised this study. All authors contributed to the article and approved the submitted version.

## Supplementary material

Supplementary Figure 1 Berry phenotype and physicochemical parameters from different sampling stages under different treatments. **(A)** Berry phenotype. Berries were collected at DAA 30, 50, and 70, taken by Canon camera (TKY, JPN). **(B)** External and internal quality parameters, including longitudinal diameter, transverse diameter, single berry weight, total soluble solid (TSS), and titratable acids (TA).

Supplementary Table 1 Primers for qRT-PCR. The sequences of key genes in BR/JA biosynthesis and aroma substances biosynthesis pathways were shown in the table.

Supplementary Table 2 The information and retention time of free monoterpenes detected in this study.
