# Supplementary figures and images for: EBR and JA regulate aroma substance biosynthesis in ‘Ruidu Hongyu’ grapevine berries by transcriptome and metabolite combined analysis
Source: Front Plant Sci. 2023 Jun 6;14:1185049. doi: 10.3389/fpls.2023.1185049 (PMC10279965; doi:10.3389/fpls.2023.1185049)

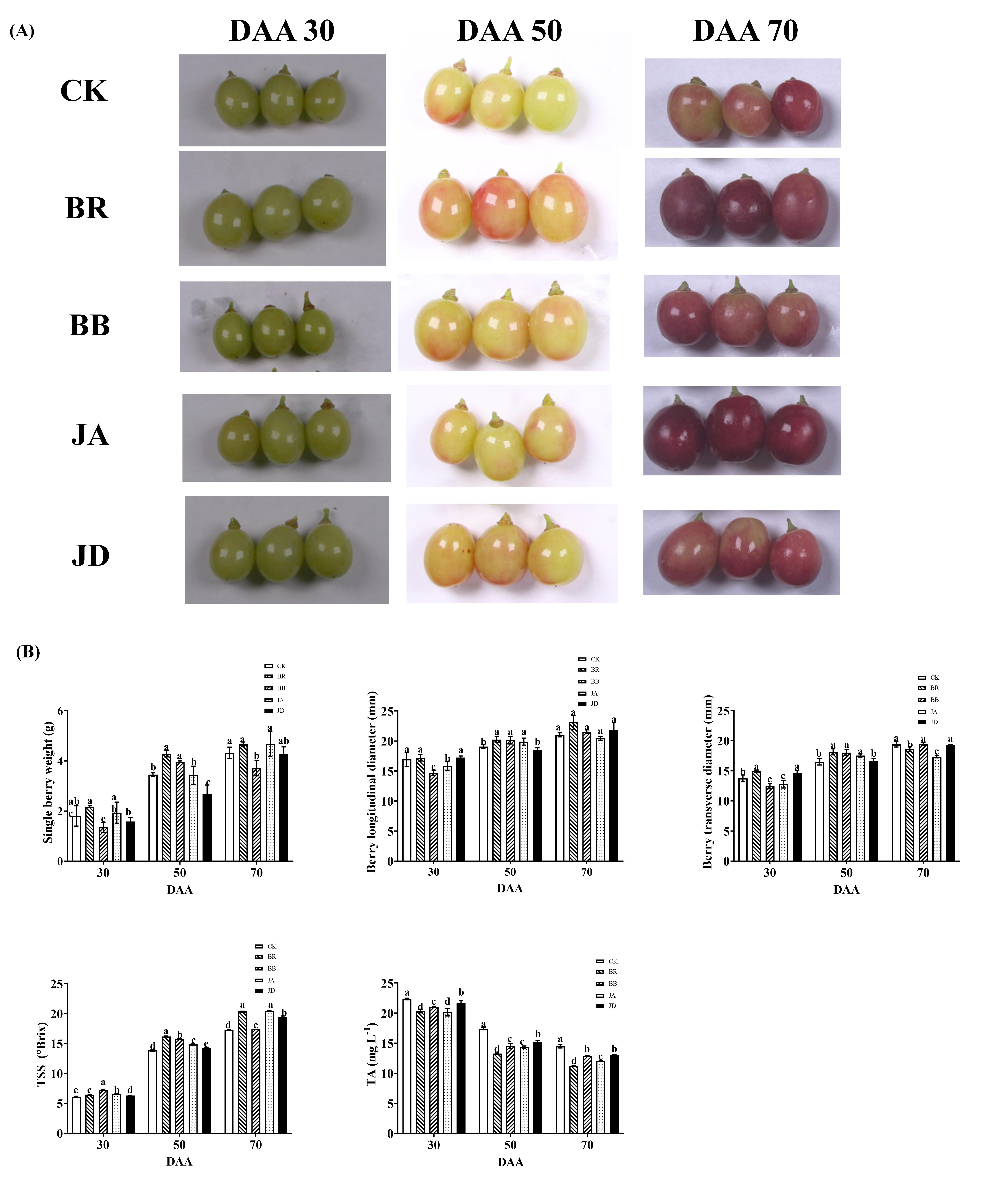

Supplement: Supplementary Figure 1 — Berry phenotype and physicochemical parameters from different sampling stages under different treatments. (A) Berry phenotype. Berries were collected at DAA 30, 50, and 70, taken by Canon camera (TKY, JPN). (B) External and internal quality parameters, including longitudinal diameter, transverse diameter, single berry weight, total soluble solid (TSS), and titratable acids (TA). [file Image_1.jpeg]
