# Supplementary material for: EBR and JA regulate aroma substance biosynthesis in ‘Ruidu Hongyu’ grapevine berries by transcriptome and metabolite combined analysis
Source: Front Plant Sci. 2023 Jun 6;14:1185049. doi: 10.3389/fpls.2023.1185049 (PMC10279965; doi:10.3389/fpls.2023.1185049)
Supplement: Supplementary Table 1 — Primers for qRT-PCR. The sequences of key genes in BR/JA biosynthesis and aroma substances biosynthesis pathways were shown in the table. [file Table_1.docx]

| **Target genes** | **Forward sequence（5’ → 3’）** | **Reverse Sequence（5’ → 3’）** |
| --- | --- | --- |
| *Vvactin* | GACAATTTCCCGTTCAGCAGT | GATTCTGGTGATGGTGTGAGT |
| *VvAACT (VIT_00s0531g00050)* | AGTGAGTACGGTGATAGAGTGA | CGATCAAATTTGTACGACTCCC |
| *VvHMGS (VIT_02s0025g04580)* | CATTGCTGATGCTGACTACTTC | GCATCATATAGTGGCTTTGCAA |
| *VvHMGR (VIT_03s0038g04100)* | TCGATTACGAGTCGATTTTGGG | GGAGTACTCTCTTCCATCCAAC |
| *VvPMK (VIT_14s0128g00330)* | AGATTTGCGTTGGTAGTTGTTC | GTACACAGAAACACAACAGCAT |
| *VvFPPS (VIT_19s0015g01010)* | AGTTTTTCTTGCATGTGTCCTG | GGCTTTCCTTTGAAATGGTTCT |
| *VvGPPS (Vitvi10g04224)* | AGCTTTAAATGTGCCTCTACCT | ATTGTTGTCTTGTGCGTAGTTC |
| *VvTPS (VIT_02s0012g01680)* | GAAGAAGATATCTCGAGTGGCA | GGTTTCCCACAATTATCATCCG |
| *VvDXS (VIT_05s0020g02130)* | GGTGGTGCATGATGTAGATTTG | CAAGCCATGAAAGTGACATCAA |
| *VvDXR (VIT_17s0000g08390)* | ATATGGGGAAAAAGATCACGGT | CATCATATTCAGCCCCAAACAG |
| *VvMCT (VIT_14s0060g01920)* | AACATGCATTACCTCAATGACG | GATACGGTGAAAACTGCAGATC |
| *VvHDS (VIT_06s0004g02900)* | ATGGGTTGCATTGTAAATGGAC | CCATAGCAATTCCTCGCTTTAC |
| *VvGGPPS (VIT_05s0020g01240)* | CAATTTCTGATGGAAAGGGAGC | TAAAGATTGACTCCGCTGAACT |
| *Vv13-LOX (VIT_13s0064g01490)* | CTAAACTTCGGCCAATATCCCT | GTTGAGGAAGTTGGTGTATTCG |
| *Vv12-HPL (VIT_12s0059g01060)* | GGAAGAGGATCGACAAGTACAA | GCGATTACGTTAGGATTGACAC |
| *VvADH (VIT_18s0001g15410)* | CAAAAAGATACGAAGACGCCAA | GGTCATCTCCACAATAACCTCT |
| *VvISO (VIT_03s0017g01060)* | ATGGGGAATACAACCTAACTCG | ATAGGCTTCTCATCCAAACCAA |
| *Vv9- LOX (VIT_09s0002g01080)* | CTAAACTTCGGCCAATATCCCT | GTTGAGGAAGTTGGTGTATTCG |
| *Vv13-HPL (VIT_03s0063g01820)* | CCATAAGGGATCGTTTCGACTA | CTCTTCTCGACTTTGGAAGAGT |
| *VvDWF4 (VIT_04s0023g01630)* | CTGAAGCATTCCAACCTTTCAA | GCCTTCCAAGAAGTAGATAGCT |
| *VvBR6OX1 (VIT_14s0083g01110)* | TTCCATGATGGCTGTCAAGTAT | CGCATCAACTTGTAGTCATTCC |
| *VvBR6OX2 (VIT_01s0011g00190)* | CAGAAAGATTGATCAGGGCATG | GGCTGATTCAGTTTCCATGATC |
| *VvBAS1 (VIT_18s0001g12200)* | ATTGCAGCATGACATACTTCAC | CAAGGGGTTAAACCTAGCCTTA |
| *VvDET2 (VIT_08s0007g01760)* | TCTGTACACATGTGCCAATTTG | TGGTACATCGTCATTCACTTGA |
| *VvCPD (VIT_13s0067g00660)* | GAAGAGGAGAAGAAGAACGACA | TTATCCTTGCCCTAATCTCGTC |
| *VvROT3 (VIT_04s0023g02650)* | GATTATGCCTGGACTGACTACA | TTAACAGCTTTTCTCCAAACCG |
| *VvLipase (VIT_12s0059g01550)* | ATTTGTAATAAACCGGGCATCG | GGGATGTTCGTCATGTTGTATG |
| *VvLOX (VIT_14s0128g00780)* | ACAACTTCCACGAAAACTTACG | GAGTCATTCACAGCAGCATAAG |
| *VvAOS (VIT_03s0063g01850)* | AAGTCGAGAAGAGAAACGTCTT | GTAGAGAGAAGGAGAAGCGTTT |
| *VvAOC (VIT_14s0083g00110)* | GCCACATACAGCTTCTATTTCG | AGATACGTATCCTCGTAGGTCA |
| *Vvopr3 (VIT_11s0016g01230)* | TCAAACCGGTGCAAATTCTTAG | GTGATCAATTGCTGGTGAAACT |
| *Vvopcl1 (VIT_01s0010g03720)* | TGTGCACCAGAATGATCTATGT | ATGTGAAAGAATGGCATTAGGC |
| *Vvacx1 (**VIT_00s0662g00010)* | CTTCGCAAGCATTCTAAGACTC | GCAACATAATCTACAATGCGGT |
| *Vvacx5 (VIT_05s0020g01740)* | CGCTCCTTCGTCAAAGATTTAC | CCAAGATACCAGACTATGACCC |
| *VvJMT (VIT_18s0001g12900)* | GAGCTGGAACAAGGGAAAAATT | CACTATTTCTTCTGCACGTGAC |
| *VvMJE1* | ATGGAGAAAAGAGAGAGGCA | CTCTCCATAGCAACCGATACG |
| *VvJAR1 (VIT_15s0046g01280)* | GAATCTTCTGCTGACGATCAAC | CCAGGATCAGTCGATTTATCCA |
| *VvJIH1 (VIT_18s0001g02570)* | CGGACTGCACAATTTGGATTAA | GGTATTGAATTCCTCGAAAGCC |
