# Supplementary material for: EBR and JA regulate aroma substance biosynthesis in ‘Ruidu Hongyu’ grapevine berries by transcriptome and metabolite combined analysis
Source: Front Plant Sci. 2023 Jun 6;14:1185049. doi: 10.3389/fpls.2023.1185049 (PMC10279965; doi:10.3389/fpls.2023.1185049)
Supplement: Supplementary Table 2 — The information and retention time of free monoterpenes detected in this study. [file Table_2.docx]

| **Terpenes substance** | **Retention time (min)** |
| --- | --- |
| *Myrcene*  *Trans-rose oxide*  *cis-3-Hexen-1-ol*  *Trans-Linalool oxide*  *1-Octen-3-ol*  *Nerol oxide*  *1-Hexanol, 2-ethyl*  *Vanillin*  *Linalool*  *Hotrienol*  *Crotyl alcohol*  *Terpineol*  *Neral*  *Vanillyl Alcohol*  *Nerol*  *isogeraniol*  *Geraniol*  *Geranic acid* | 5.2  11.8  13.2  15.4  15.8  16.3  17.2  18.5  19  21  24.2  24.8  24.68  27.4  28.6  29  30.45  45.5 |
